# Let the Chips Fall! Public Nudging Arrangements, Coercion, and the Role of Independent Shopkeepers

**DOI:** 10.1007/s12115-023-00844-x

**Published:** 2023-05-12

**Authors:** Matti Häyry, Johanna Ahola-Launonen, Tuija Takala

**Affiliations:** Aalto University School of Business, PO Box 21210, FI-00076 Aalto, Finland

**Keywords:** Nudging, Libertarian paternalism, Public health policy

## Abstract

Nudging, according to its inventors and defenders, is supposed to provide a non-coercive way of changing human behavior for the better—a freedom-respecting form of “libertarian paternalism.” Its original point was to complement coercive modes of influence without any need of justification in liberal frameworks. This article shows, using the example of food-product placement in grocery stores, how this image is deceptive. Although nudging *practices* may not restrict the freedom of consumers, nudging *arrangements* by public health authorities do restrict the freedom of shopkeepers in standard liberal senses. Libertarianism cannot justify this coercion, and the creed is best left out of the equation as the ideological ruse that it, in this discussion, is. Other liberal theories can justify the coercion, but on grounds that can also be applied to other methods of public health promotion by subsidies and regulation. This result reaffirms that nudging should be seen to complement, not to replace, those other methods.

## Nudging—the Promise and the Issues

Nudging has been hailed as libertarian paternalism—a form of changing human behavior for the better without coercion, or without interfering with people’s freedom (Thaler and Sunstein 2003). Our aim in this article is to show that while the practice has its merits, the application of the concept in certain standard business contexts involves a coercive element that has not been explicitly accounted for in the existing literature. The coercion involved can be justified, but not by the leading arguments that have been used in legitimizing nudging. This is not fatal for the practice, but it confirms that the expectations laid on nudging arrangements as a libertarian form of paternalism are misplaced. We suggest that a proper understanding of the coercive element and its justification legitimizes a wider range of public measures that are needed in combating the covert and detrimental pressures of the market. This is in keeping with the original presentation of the idea, which was to introduce the new instrument as a complement rather than a substitute for other methods (Thaler and Sunstein 2003; Loewenstein and Chater 2017; Thaler 2017).

Nudging is a mechanism of changing human behavior for the better. Our choices are always influenced and sometimes dictated by a combination of internal and external factors. When it comes to suboptimal choices, these factors can include lack of knowledge, lapses in self-control, cultural biases, and the easier accessibility of some alternatives than others, to name a few. Nudges can target all these, especially accessibility. They are intended to change the “choice architecture” facing people so that it would be easier for them to make better decisions. Richard Thaler and Cass Sunstein stated in their seminal work (2008) that “better” in this equation means choices that people would genuinely want to make anyway, only they are impeded by their own “behavioral biases” and the kind of choice architecture that amplifies these biases. The nudged decisions typically aim at people’s own good, but the well-being of others or environmental considerations may also provide the motivation (Schmidt and Engelen 2020).

An oft-cited example, and the one we use, is the placement of food products such as potato and other chips which are low in good nutrients but rich in sugar, fat, and calories—colloquially titled “junk food”—in shops and grocery stores. Studies have shown that sales are higher for items placed on eye level and on checkout lanes (Thaler and Sunstein 2008; Just and Wansink 2009). Giving the visibility advantage to fruit and vegetables instead of junk food can make consumer choices healthier.

The concept of nudging was defined by Thaler and Sunstein to introduce a non-coercive, freedom-respecting alternative to prohibitions, commands, restrictions, and economic incentives: “Our goal [is] to defend libertarian paternalism, an approach that preserves freedom of choice but that authorizes both private and public institutions to steer people in directions that will promote their welfare” (Thaler and Sunstein 2003, 179). The idea in the junk-food placement case is to guide consumers toward healthier alternatives like fruit and vegetables by changing the way in which options are presented to them. The (dual) justification, in a nutshell, is that health risks are reduced (a good effect) without interfering with the freedom of individuals (absence of bad effect) (Thaler and Sunstein 2008).

Extant literature abounds with criticisms and defenses of Thaler and Sunstein’s view. We limit ourselves to presenting a concise outline of them. This is meant to be indicative of those concerns in the field that are relevant to our endeavor, not a full-fledged survey that can be found in many of the sources we cite.

The criticisms leveled at nudging have concentrated on the practice’s power to manipulate our choices for business and political ends that are not necessarily our own. We do what the nudgers want us to do (Hausman and Welch 2010, 128); our desires and actions are no longer autonomous (Schmidt and Engelen 2020); our rational and moral agency is violated (Bovens 2009; Grüne-Yanoff 2012; Conly 2013, 30; MacKay and Robinson 2016); and governments assume a power to exercise unsavory domination over us (Hausman and Welch, 2010; Jones et al., 2011; Grüne-Yanoff 2012).

Defenses against these criticisms include that nudges are so effortlessly resistible (Saghai 2013, 489) or so “easy and cheap” to avoid (Thaler and Sunstein 2008, 6) that there is no actual infringement of autonomy. One indication of this is that removing correctly devised nudges would not enhance or restore the autonomy of the nudgees (Engelen 2019). On the contrary, the presence of nudges can promote autonomy by helping the nudgee to “choose according to her own conception of the good in situations where she would have previously done otherwise” (Mills 2015, 503). Also, since behavioral biases are undeniably real, respecting people as full agents cannot be limited to respecting them as perfectly rational and autonomous decision-makers. This would mean protecting a theoretical idealization, not an empirical entity (Engelen 2019; Schmidt 2019). Besides, choice architectures already exist and they have come into existence somehow, based on some interests. What, then, could be wrong with reshaping them to promote people’s health and well-being (Pilaj 2017, 751)? Democratic nudging by public policy can in fact counteract the feared detrimental domination, which precedes deliberate health and related nudges. The original, uncorrected choice architecture has been created by shopkeepers and grocery-store chains to maximize profits, with little or no consideration of what is good for us, anyway (Schmidt 2017).

## Our Tasks, Approach, and Conceptual Framework

The criticisms and countercriticisms of nudging are not always directly comparable. They are based on different conceptual and ideological presuppositions, and often “speak in different languages.” Our first substantive task here (in the section “Political Moralities and Freedom”) is to make this visible. We utilize a “map of justice” which has been produced to clarify normative divides in bioethics (Häyry 2018; 2022a) and applied to several topics and fields, including moralism in healthcare (Ahola-Launonen et al. 2019; Häyry 2019), solidarity during the COVID-19 pandemic (Häyry 2022b), and the possibility of a sustainable bioeconomy (Häyry et al. 2021; Häyry and Laihonen 2022). Conceptions of freedom and views of justice form alliances that explain (our next task) some incompatibilities in the condemnations and vindications of nudging and nudging arrangements.

The analysis of the presuppositions will pave the way to our second substantive task, which is to expose the coercive element in public nudging arrangements (in the section “Nudging and Freedom”). The point as such is not difficult to make. Thaler and Sunstein’s (2008, 6) pivotal definition confines the scope of nudging as follows:

A nudge, as we will use the term, is any aspect of the choice architecture that alters people’s behavior in a predictable way without forbidding any options or significantly changing their economic incentives. To count as a mere nudge, the intervention must be easy and cheap to avoid. Nudges are not mandates. Putting fruit at eye level counts as a nudge. Banning junk food does not.

The notable point here is that the role of nudgers is assigned to shopkeepers. They are the ones who are supposed to alter consumer behavior by changing the choice architecture. They are the ones who are supposed to place fruits and vegetables, not junk food, at eye level. But why would they do this? The reasons could include a commitment to the customers’ health, trust in nutritional science, and a belief that making their stance known will optimize returns (Houghtaling et al. 2019). If none of these reasons apply, however, something else is needed. Regulation that prompts shopkeepers into becoming nudgers is then the most obvious answer. This changes the situation insofar as freedom and coercion are concerned, though. The relationship between shopkeepers and their customers (and between the state and its citizens) remains freedom-respecting, but the relationship between public authorities and shopkeepers becomes coercive.

This is not by any means a lethal blow to nudging or related practices. Voluntary changes in choice architecture are still legitimate; and since all political moralities allow coercion under some circumstances, justifications for related practices can be found in many ideological directions (Häyry and Takala 2015). It is just that fully uncoercive nudging arrangements as a systematic practice would require that most shopkeepers decide, independently and without pressure, that they wish to rearrange their shops. Otherwise, wider nudging arrangements would need regulations steering product placement, which do not seem to fall under the original concept at all. The introduction of nudges was supposed “to defend libertarian paternalism, an approach that preserves freedom of choice” (Thaler and Sunstein 2003, 179). Nudges were meant to be so easy to resist that they cannot present a risk to anyone’s autonomy (Saghai 2013, 489). This is not always the case with state attempts to change consumer choices. Thaler and Sunstein’s own example of non-nudging was banning junk food, and their focus was on the freedom of consumers. Shift the attention to the shopkeepers, however, and the situation changes. Now *their* choices are clearly restricted. Our third major task (in the section “Public Nudging Arrangements and Coercion”) is to clarify the ways in which business people’s freedom can be thought to be limited by product-placement regulations and to highlight the justifications which different theories of justice can offer for them.

Our fourth and final major task (in the section “Legitimate Coercion Beyond Nudging”) is to apply the results of the examination (of the previous sections) to other health-promoting public measures. If coercive nudging arrangements are justified, then the same logic can presumably be applied to other forms of state steering. Depending on the background theory, the legitimation of coercion can be based on beneficence (promoting well-being), non-maleficence (preventing harm), autonomy (preserving or enhancing individual freedom and self-rule), good practices (communal or political, conservative or emancipatory), protecting private property, combatting alienation, and other constituents of a good and fair social life. Some of these are compatible, others in conflict with one another. Our investigation into them will reveal their mutual relations, strengths, and weaknesses.

Before plunging into theories of justice as expressions of political morality, and their interconnections with cognate concepts such as “freedom,” “coercion,” “autonomy,” “choice,” and “best interest,” however, a word concerning our approach and theoretical framework is in order. In short, ours is an exercise in applied moral and political philosophy. We apply moral and political concepts to an archetypal case of nudging to test the intuitions behind a popular interpretation of an emerging policy and practice. The intent is critical and the knowledge interest is emancipatory. The moral and political concepts that we employ are ideal types which are explicated as the narrative progresses. The words used mean what we postulate them to mean and, for the economy of the presentation, no comprehensive engagement with other linguistic usages is involved. Nor do we engage with the innumerable varieties of nudging in the real world of policy and practice. As philosophers, we deal with the categories of “all,” “some,” and, as a specification of the latter, “there is a case.” Our case is the one presented by the originators of the theory, the placement of junk food, and no empirical quantification is included. As a result, our conclusions are conceptual and, at best, assertively hypothetical (Häyry 2015; Räsänen and Häyry 2022).

## Political Moralities and Freedom

Theories of justice (or ideologies or political moralities—we use these expressions interchangeably) come in different packages depending on their background assumptions, including the concept of freedom or liberty they rely on or generate. Almost everybody agrees that the core of justice is equality. We have equal worth; our interests should be respected equally; in political life, everyone should be counted as one and no one should be counted as more or less than one; and we should be heard, or taken into account, in public decisions that affect us. After this preliminary agreement, however, views diverge (Häyry 2018; 2022a; b). To understand the normative dimensions of the discussion on nudging and nudging arrangements, let us map the main views involved and their key presuppositions.

Some believe that justice centers on the private control of property, particularly means of production, and on an adjacent conviction that individuals are themselves responsible for their own happiness and well-being. Others hold that it would be better to have the means of production in some kind of public control, and that we should assume a shared responsibility for each other’s fates. Some are committed to the idea that norms and values are universal, or the same for all. Others prefer the stance that norms and values are positional, or dependent on their holder’s situatedness in intersectional relations. Some believe that justice would be done by catering to global needs, directed by calculations and social engineering based on measurable units of objective good. Others hold that justice is a matter of tending to local interests, guided by spontaneously shaped practices and traditions.

**Fig. 1:**
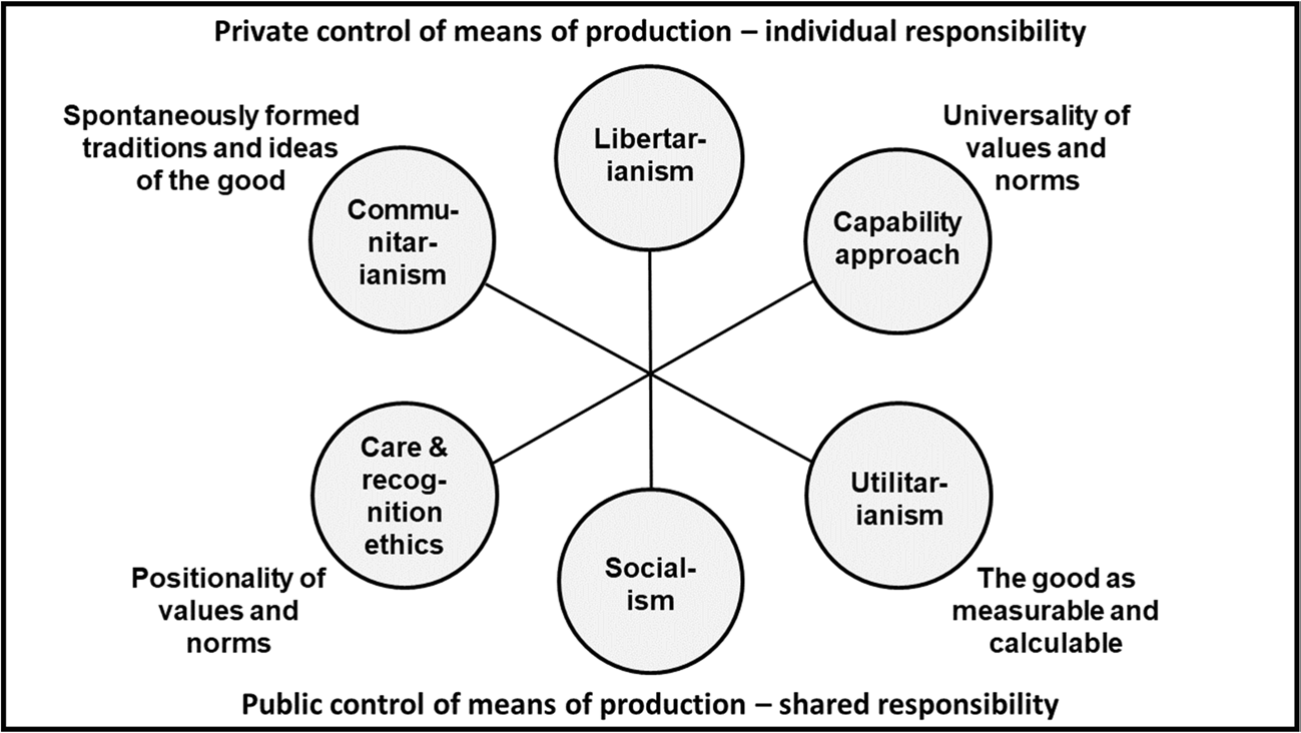
Political moralities and their main background assumptions on a map of justice

Figure 1 presents schematically the six views that these polarities define.

The theory of justice stressing the importance of private property is *libertarianism*. Robert Nozick’s *Anarchy*, *State*, *and Utopia* (1974) expressed it decisively. Individuals have rights to their life, bodily integrity, and possessions against deliberate violations by others. These rights can be justified by a fictional reconstruction. Those interested in their own life, limb, and belongings establish a dominant protective agency to shield themselves against each other and against outsiders. When the rule of the protective agency is extended beyond the original signatories, others are deprived of their right to defend their own ideas of justice in their own way, but this is compensated by the equal protection the agency provides for their life, person, and property. This is the proper limit of state power. A minimal night-watchman state safeguards its citizens’ rights to life, bodily integrity, and property against other agents, but goes no further. Educational, social, and cultural services do not belong to the state’s legitimate remit, because running them would require redistribution, and this would violate property rights without adequate compensation. This is the theory that allegedly underlies the nudging ideology.

At the opposite end of the property continuum is theoretical *socialism* as formulated by Karl Marx and Friedrich Engels (2004 [1848]). Their ideas have not been fashionable of late, but in the discussion on freedom they are indispensable. This is another fictional narrative. Capitalism is an economic and political arrangement that requires continuous growth. When populations and economies grow, consumerist well-being is spread more and more widely. At some point, however, humanity runs out of new technologies and natural resources, and they cannot be exploited to drive growth anymore. Only the workforce remains available, and it is increasingly exploited. This completes the alienation of the workers from the products of their labor, from each other, and finally from themselves as full human beings. Becoming aware of this, workers unite and overthrow the capitalist system. What happens next is not clear. Marx and Engels had their hopes of a stateless society, but reality has not lived up to their expectations. Anyway, the socialist defense of nudging could be based on a heightened alertness concerning the class-related implications of choice architectures based on capitalist logic and power relations.

The universality of norms and values is central to many liberal stances. The tension with positionality is at its most visible in the *capability approach*. Amartya Sen (1992; 2011) and Martha Nussbaum (1998; 2006) have developed this view. The starting point is that individuals may prefer courses of action that they, due to structural oppression, falsely identify as the best for themselves. These “adaptive preferences” should not be the measure of public policies. Instead, the yardstick should be people’s ability, capacity, and opportunity to do and to achieve things that are important to their human flourishing. When a young woman decides to remain in her native village and assume the traditional “woman’s role” in the community instead of seeking an education or employment in the city, her preference can be adaptive. Enhanced provision of available opportunities by the public authorities could then be used to bypass the suboptimal choice. Sen has argued that these situations must be assessed in their own contexts, and that universally desirable capabilities cannot be defined once and for all. Nussbaum (2006, 76–79) has devised a much-used list of ten central human capabilities. Although nudging is not about altering preferences, Thaler and Sunstein’s (2008) definition of the goal of nudging bears a close resemblance to the capability approach in that it is to facilitate decisions that would be genuinely beneficial for the person.

The modern move toward positionality in norms and values began with Carol Gilligan’s *In a Different Voice* (1982), and its roots are in feminism. Studies in social psychology had suggested that the apex of moral development is reached in rule- and consequence-based ethics. The study subjects, however, had been exclusively boys and men. Gilligan investigated girls and women and came to the conclusion that there is a further level, a level of recognizing and cherishing care and special relationships. Her focal case was the relationship between a mother and a child. The recognition of gender differences and intersectional identities became increasingly pronounced in the works of Iris Marion Young (1990), Judith Butler (2016), Donna Haraway (2016), Kimberlè Crenshaw (2019), and Patricia MacCormack (2020). The common theme of these diverse takes on *care and recognition ethics* has been that principles and outcomes serving only certain groups in power should be augmented or replaced by more inclusive and less hegemonic concerns. This reflects the idea of care and recognition to groups that are more vulnerable to product placement.

Universal well-being, calculations, and social engineering form the core of *utilitarianism* in its many guises. One of the main differences between the versions is the definition of what is good. The doctrine states that all our actions and decisions should be aimed at the greatest good of the greatest number and that everybody’s good should be taken into account with equal weight. What is meant by “good” varies, though, from simple pleasure and absence of pain through higher forms of pleasure and cultural enjoyment to qualified preference and desire satisfaction (Häyry 1994; 2021; 2023). Two useful definitions in the context of nudging connect the good with rational preferences and rational desires. John Harsanyi (1982) developed utilitarianism as a semi-economic theory and suggested that public decisions should be based on maximizing the free, informed, and uncoerced preference fulfillment of the citizenry. Richard Brandt’s (1979, 11–12) ideal was to maximize the satisfaction of rational desires—desires held by a person who has undergone “cognitive psychotherapy” and whose desires have been “maximally influenced by evidence and logic” (Brandt 1979, 11). Both ideas are close to the “non-adaptive” preferences take of Sen and Nussbaum’s capability approach, and hence Thaler and Sunstein’s view on value—good is defined by the genuine wish of the person.

Those who believe that justice is a matter of protecting local interests and traditions can find a theoretical home in *communitarianism*. Utilitarian calculations and social engineering are, they can argue, wrong because such maneuverings disrupt the spontaneously, historically, and communally formed ways of life that make people’s lives sociable and good. Michael Sandel (1982; 2009) has stressed the importance of solidarity and the relation between the self and the community. Values and conceptions of the good are formed through the community, not by a rational self. What makes people’s lives good is a sense of belonging. If we start believing that everything is in our own control, we stop seeing our interconnectedness with one another and our inability to cope without collaboration with others. Our feeling of solidarity can then be lost and we can slide into the abyss of consumerist individualism. The way to prevent this is to protect the community’s moral and civic goods from the vices of the commodifying market economy that rather separates than unifies people (Sandel 2007; 2013). This view can support nudging, if its ends and means are appreciated as common goods, or condemn any attempts to alter existing choice architectures, if the changes would go against valued traditions. The latter is the case at least with conservative (as opposed to “responsive”) communitarianism (Young 2020).

How we understand freedom and liberty depends on our political morality. For *libertarians*, freedom means that other people and institutions do not actively interfere with our private property. For *socialists*, it means that we (the proletariat or the precariat) liberate ourselves from the yoke of the capitalist economic system. The contrast is stark. One view is based on an atomistic individualism, where historically accidental property holders are in need of protection against the needs of their antagonistic fellow beings. The other rests on a collectivist understanding of human interdependencies, according to which the “individual” is a hegemonic construction that perpetuates the *status quo*, with its related inequalities. It is no part of our analysis here to take sides between the views. We are simply listing these possibilities in order to bring clarity to the discussion on nudging and nudging arrangements.

For *capability ethicists*, the stress is on “positive freedom,” or “freedom to” as opposed to the “freedom from” interference favored by libertarians. As noted, this, rather than pure libertarianism, is the notion that Thaler and Sunstein seem to lean on—but more on that in the next section. It should be stressed here, by the way, that the divide between “from” and “to” in these two creeds is *not* the classic distinction between the two concepts of liberty introduced by Isaiah Berlin (1969 [1958]). His demarcation sets apart the “negative” (individualistic) absence of interference from others and the “positive” (collectivist) self-realization through joint political action. The reference point is, rather, Joel Feinberg (1973, 4–19), who situated both the “from” and the “to” approaches in a liberal framework. In his model, negative freedom means the absence of obstacles in the path to our destination while positive freedom means the presence of factors that enable us to complete the journey. For *care and recognition ethicists*, individual freedom is not paramount in the sense that it is to “atomistic” liberals like libertarians, utilitarians, and capability theorists. They argue that equality of opportunity as an expression of positive freedom should be secured to members of intersectionally crossing oppressed groups. This is more in keeping with Berlin’s demarcation.

For *utilitarians*, freedom has traditionally meant nonrestricted options (Driver 2014). Unlike libertarians and capability theorists, however, they do not see freedom as an intrinsic value, to be protected for its own sake. It should be defended, according to them, but only insofar as it is instrumental to the well-being of individuals and to the flourishing of economic and social life. When it is not, its status has to be reevaluated. The difference between utilitarians and capability theorists is a fine line—but again, more on that in the next section. For *communitarians*, the freedom of individuals as separate entities is not on the agenda to begin with, because individuals as separate entities are not the primary objects of protection. Personal freedom as an entitlement to denounce the culture into which one is born is not of high value among conservative communitarianism.

## Nudging and Freedom

Since there are many notions of being free (and since it is not our aim here to prioritize any of them), the question is not, “Does nudging restrict freedom?” but, “In which senses does nudging restrict freedom and in which senses not?”

Those who argue that the practice as such forces us to do what businesspeople and politicians want us to do (Hausman and Welch 2010) can employ either a “negative” utilitarian or a “positive” liberal concept of freedom. If our actions are restricted every time an option—say, the option of easily choosing junk food—is eliminated, then nudging in the way suggested by Thaler and Sunstein curbs our freedom. This does not necessarily mean that utilitarians or capability theorists would condemn the practice. It can still guide people into doing what they would have done freely, informedly, autonomously, and non-adaptively (Mills 2015, 503). When businesspeople and politicians nudge us into this direction, all should be well. It is, however, possible that they guide us into other directions for other reasons (Thaler 2015). If this is the case, and if their nudges clash with our genuine preferences, the situation is different. The justification must then be sought separately, from the rightful grounds of restricting freedom. This is an issue that we will tackle in the next section.

Before continuing the story in the individualist liberal framework, let us take a look at a challenge that relies on the collectivist positive notion of freedom and claims that manipulation as such is wrong, whatever its motives. This tallies with the concerns that nudging renders our desires and actions nonautonomous (Schmidt and Engelen 2020), that it violates our rational and moral agency (Bovens 2009; Grüne-Yanoff 2012; Conly 2013; MacKay and Robinson 2016), and that it allows governments to exercise illicit domination over us (Hausman and Welch, 2010; Jones et al., 2011; Grüne-Yanoff 2012). These are all legitimate conceptual concerns, and none of them can be exhaustively rejected by the liberal insistence that nudges are easy to resist (Saghai 2013, 489) and to avoid (Thaler and Sunstein 2008, 6). However innocuously, the nudges, and the power structures that have generated them, are nonetheless there and contribute to a choice architecture that may hinder the realization of higher goals such as heightened class awareness, recognition, and exposure of hegemonies, or respect for traditions that uphold communities. In these cases, they would surreptitiously violate the ideas of freedom promoted by socialists, care ethicists, and communitarians.

Arguing that behavioral biases (Engelen 2019; Schmidt 2019) and choice architectures (Pilaj 2017, 751; Schmidt 2017) preexist any deliberate nudges does not help, either. Socialists and care ethicists can maintain that nudges are such cosmetic corrections that they merely halt historical progress toward revolution or recognition by creating a veil of mist that covers the capitalist, surreptitiously coercive agenda. Conservative communitarians, for their part, can resist any tampering with choice architectures that are part of “our” lifestyle. More revisionist views can, of course, support nudges as an improvement to community tradition or the lives of the oppressed. However, none of these challenges the core of the nudging ideology. The objections are derived from views that rely on core values other than individual freedom. In this sense, they are *external* criticisms against a liberal practice, and hence something to be expected, when differences in political moralities are taken seriously, like they are in our map of justice. The *internal* challenge to Thaler and Sunstein, though, is the shopkeeper.

To elaborate on this, let us return to liberal thinking, Thaler and Sunstein’s own ideological terrain. The story there has two sides. Nudging by placing fruit and vegetables instead of junk food at eye level does not raise any objections in the libertarian, capability, or utilitarian camps. The customers’ property rights are not violated, the opportunity to make one’s own choices to pursue one’s own ends is not threatened, and while an (unhealthy) option is removed, the good of all is probably served by doing so. With these observations, the *practice* of nudging is exonerated. Not so, however, with wider nudging *arrangements*. This is where the shopkeepers enter the picture. They could, in theory, be motivated to be health nudgers by philanthropy and science, or by a belief that it will improve their returns (Houghtaling et al. 2019), but this is not always the case. If anything, businesspeople tend to use nudges to increase their own profits without thinking about their customers’ well-being. As Thaler (2015) has warned, this is bad nudging and should not be engaged in.

To hammer home the improbability of the first motivation, it can be put in the form of an Aristotelian practical syllogism for a virtuous person: “I desire my customers to be healthy and happy. I believe that my customers cannot be healthy and happy unless I place fruit and vegetables instead of junk food at eye level. Therefore, I place fruit and vegetables instead of junk food at eye level.” The belief part of this can be influenced by public arrangements that do not restrict freedom, for instance, by information campaigns directed at shopkeepers. The desire part is trickier, not because shopkeepers would want ill-being or misery to their customers, but because they may want other things in addition to client health—well-being for themselves, success in business, and economic prosperity. Their practical syllogism can then take other forms.

This leaves the possibility that being a health nudger would produce better returns to the shopkeeper. To persuade shopkeepers to change their ways, they should be convinced that responsible business in this particular sense would in their case also be profitable business. This can be easier said than done. General studies into the connection between responsibility and profitability are divided (Hermawan and Mulyawan 2014; Wang 2014; Mikolajek-Gocejna 2016; Barauskaite and Streimikiene 2021), and they concentrate on corporations, investments, and other “big issues.” The evidence is not sufficient to assure shopkeepers that health nudges would optimize their returns. Benevolent lying by the government (about the profitability) is not acceptable, either, at least not according to Thaler (2015), who contends that nudges should always be honest and transparent. The only option open to public authorities who want to promote population health in this way, then, seems to be restricting the freedom of independent shopkeepers.

We are talking about “independent shopkeepers” here, although the concept has its ambiguities. Most merchants are somehow connected to wider chains and answer to them, so their product placement can be dictated or directed from above. When the connection is tight, restrictions of freedom can appear on two levels: public health authorities regulate chains and chains control their retailers. We concentrate, for the time being, on the relatively independent shopkeepers who can themselves make their decisions to nudge or not to nudge.

The reasons for *not* becoming a health nudger can be quite mundane (cf. Dholakia 2016; Houghtaling et al., 2019). “My customers are used to the chips being where they are.” “Sure, I can sell them more fruit and vegetables, but will they be eaten or will they just rot in their fridges?” “Is it really my business to intrude in my clients’ affairs?” “And why should I do what some know-it-all scientists suggest, anyway?” Facing these objections, public health authorities are left with a choice. They can provide more information in the hope that something changes. Or they can lure shopkeepers into nudging by subsidies. (More on this in the final section.) Or they can regulate. If the last alternative is chosen, shopkeepers will probably be coerced, at least in senses assumed on the liberal side of our map of justice (in Fig. 1). Let us take a closer look at the situation.

## Public Nudging Arrangements and Coercion

Public regulation would require the authorities to define sanctions for shopkeepers who do not place fruit and vegetables instead of junk food at eye level. This scenario does not, of course, depict the whole range of nudging arrangements, but keeping the focus on this particular case keeps things tidy and concrete for our analysis.

Within the liberal “negative” concept of freedom, the sanctions would mean that one option previously open to the shopkeepers—“Place as you like and avoid sanctions” —would be removed, and their freedom would in this sense be restricted. Within the liberal “positive” notion, there would not necessarily be any real constraint if it could be shown that the shopkeepers in question would actually prefer being health nudgers. The logic here is that the already existing choice architecture makes them mistakenly, and adaptively, believe that they want to cater to their customers’ unhealthy desires. Champions of the non-liberal “positive” idea of freedom could, in theory, argue that the state is going restrictively and coercively against a good, spontaneously formed tradition. This is at its likeliest in the conservative communitarian corner. The feeling there can be that it is not the state’s business to interfere with the junk-food culture, especially not in the name of elitist scientific concerns and social engineering. Other non-liberals could have other ideas, though.

Socialists of the non-revolutionary ilk can look favorably upon state attempts to promote public health. If the workers and the precariat have suffered ill health in the jaws of the unencumbered capitalist business endeavor, governments could actually be liberating them. The method is paternalistic, but that does not present problems to those who defend positive freedom in the name of collective good. Care and recognition ethicists of the moderate kind could easily join this bandwagon. When the public authorities work for the benefit of the vulnerable—in this case, those who are prey to the existing choice architecture—their work should not be excessively criticized. Their motivation can be calculating and their measures inadequate, but taken pragmatically, what they do is probably better than nothing. To echo the care-ethics stance, if nudges help caretakers to raise healthier children, then why not?

Somewhere between liberals and conservative communitarians, there is a notable compromise view, *republicanism*—not to be confused with any existing political parties (Lovett 2018). According to this creed, citizens of a republic are not restricted in a bad way, or “dominated,” if they are not subjected to arbitrary power. Their freedom in the negative “from” sense can be legitimately curtailed if this is based on democratic procedures and the rule of law. Liberty as non-domination is more important than the unbridled realization of individual wishes and whims. Shopkeepers could be ordered to nudge their customers without infringing their civic freedom. As responsible citizens, they should be happy to obey state regulations without complaints. It is, of course, a matter of dispute what counts as “arbitrary” power and interference. Non-republicans could argue that state regulations are, from our viewpoint, arbitrary if we have not accepted and authorized them. As retailers of a chain, we have voluntarily entered a contract that legitimizes the chain’s power over us. We have not, however, entered a similar explicit contract with the state. The republican answer to this is that political life is not about voluntary contracts but about citizenship in a well-ordered republic.

Libertarians hold a very different view. According to them, nudging regulations would be wrong in two major ways. The arrangement implies that it is in the state’s remit to tend to its citizens’ health. This is wrong, because in order to have a welfare function, the state would have to tax individuals without proper compensation, and this would be tantamount to stealing from them. Perhaps more importantly, the arrangement interferes with the way shopkeepers are conducting their business. This is wrong, because they are entitled to buy and sell what they like in whichever way they like. Call it an infringement of freedom or a violation of rights, libertarianism cannot condone nudging regulations. Paternalism is clearly in evidence, but Thaler and Sunstein’s oxymoronic expression “libertarian paternalism” cannot be reasonably extended to the arrangement (cf. Hansen 2016).

With this interim conclusion, we can leave behind the red herring of liberty-respecting and noncoercive control and move on to possible justifications for liberty-restricting nudge regulations. Our first port of call is the capability approach. Thaler and Sunstein (2008) started from the idea that nudges are meant to counteract harmful behavioral biases and choice architectures that magnify their effects. Nudging steers people into making decisions that they would themselves want to make under oppression- and domination-free circumstances. Sunstein had already in his earlier contributions (Sunstein 1994; Holmes and Sunstein 1999) flirted with capability-type axiologies (Ruger 2006). To justify the coercion of shopkeepers, however, capability theory must go beyond the notion of adaptive preferences. Health should be identified as a value that is to be promoted even when it means restrictions of freedom. But the champions of the approach are not unequivocal with such rankings. Sen’s (1992; 2011) original idea was to assess choices in their own contexts and even though health is on Nussbaum’s (1998; 2006) list, it has no clear priority over other central capabilities.

Utilitarianism offers a more straightforward solution to the justification of coercion, or the foreclosing of action alternatives by the threat of legal sanctions. The doctrine states that whenever maximum happiness or well-being or rational preference satisfaction can be produced by imposing coercive regulations, the state is both permitted and required to introduce them. Since, however, straightforward calculations may lack intuitive appeal in hard cases like the treatment of minorities (Häyry 2021), the view has been subjected to many constraining modifications (Lopez et al. 2009). John Stuart Mill (1996 [1859]) famously suggested that individual liberty is a powerful restraint and that it should not be curbed unless clear and concrete *harm to others* makes such action necessary. Joel Feinberg (1984; 1985; 1986; 1988) updated this “harm principle” in his non-utilitarian elaboration of the moral limits of the criminal law. In one form or another, it has become an integral part of the liberal canon.

In theory, this opens two routes to justifying coercive public nudging arrangements. Either show that these arrangements maximize the well-being of the population or establish that (in our case) shopkeepers harm their customers by keeping junk food at eye level. In practice, however, it would be better to employ a hybrid view. Utility calculations are notoriously complicated on this scale and stating that shop keepers clearly and concretely harm—or, to use Feinberg’s parlance, “wrong”—their clients by sticking to the unhealthy product placement can be conceptually challenged. A mere *failure to benefit* does not automatically count as a legally relevant *harm*. The hybrid proposal would say that keeping junk food visible is sufficiently harmful to legitimize regulating merchants (more on this in the final section). The public benefit is potentially considerable and the inconvenience to shopkeepers relatively small (cf. Bowie 2009 on a moral duty to nudge). Opinions on this weighing may differ, of course, and this is why our argument from here on must be conditional, of the type “If (or since) we believe that the benefits of regulation would be proportionate to its costs, we should allow and possibly encourage it.” The opposite may also be the case (Wolcott 2019).

At this point, the notion of a *reflective equilibrium* comes in handy. First introduced to choose the best rules of induction (Goodman 1955, 65–68), this meta-level principle was made widely known in John Rawls’s buildup to his theory of justice (1972 [1971], 48–51). In the model, we alternate between general axioms and more particular norms until we find a palatable balance. We can, for instance, make a considered judgment that racial minorities should have special protections, but also hold the principle that race should not influence our policies (Dworkin 1989 [1975], 29). If the view on protections is strong, the principle of racial neutrality cannot in its strictest form be our final theoretical stand. According to the doctrine of reflective equilibrium, we must revise the general rule—and possibly also our opinions on more specific norms—until the situation is stable (Häyry 2010).

The reflective equilibrium as an idea makes it possible to intellectually negotiate our way toward a compromise solution that does not attract insurmountable objections from champions of more extreme views. In a choice between two or more alternatives, the scales may tip either way, but that is not a problem for us. We are looking for a conceptual result, not a categorically binding imperative (Häyry 2015). We (whoever “we” are) can believe that coercing shopkeepers would be wrong. In this case, the idea of nudging in its original form remains intact—but public regulations concerning product placement should *not* be implemented. Or we can believe that promoting public health by regulating product placements is right—in which case governments can go ahead with this kind of coercive steering. The latter line raises, however, our final question. If nudge-related coercion for public health purposes is acceptable, could other governmental measures also be legitimized on the same grounds? And, more importantly for our inquiry, what would those grounds be in different political moralities?

## Legitimate Coercion Beyond Nudging

**Fig. 2:**
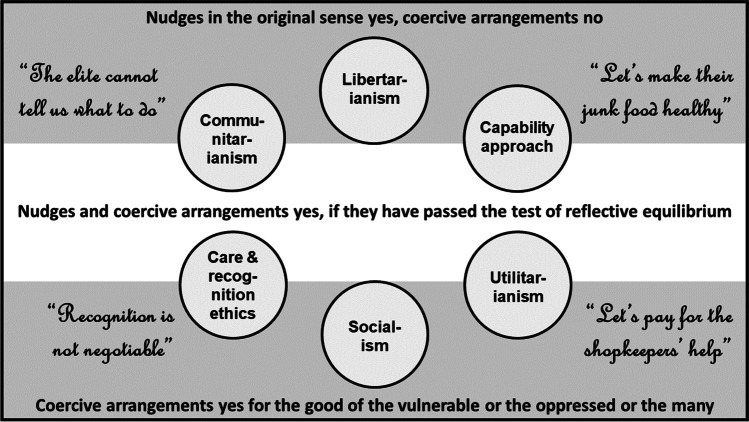
Different attitudes toward coercive arrangements on a map of justice

To answer the main question, let us return briefly to our map of justice. Figure 2 sketches how proponents and opponents of health coercion find their places in the universe of political moralities.

Libertarians are automatically opposed to public health coercion, for the reasons already cited—the state is not responsible for its citizens’ well-being and it is not allowed to interfere in the merchants’ business choices. Strict libertarians could not, in fact, condone even *noncoercive* public health nudging. That would require funding for a state health agency, governmentally supported science, and a mechanism to disseminate information. The funding would have to come from taxpayers by property-right violations.

Two other groups can join libertarians in the opposition to junk-food nudging regulations. One of them was already mentioned, namely, conservative communitarians who want to hold on to old habits in product placement and customer relations. They are not necessarily suspicious of coercion—that may be condoned in local traditions—but they may be wary of being told what to do by “elitist” public health institutes. The other group is more furtive. This is a clique that prefers subsidies and business-and-technology solutions to regulation. Well-selling comfort food should stay at eye level, its representatives say, and if customer welfare is the desired goal, the food should be modified, with state support, to be as popular as ever but inconspicuously healthy. People get what they want (a junk-food experience), shopkeepers reach their target (maximum returns), and the state gets closer to its goal (better public health). Everybody wins.

The possibility of public subsidies could also appeal to aggregative utilitarians who do not value freedom (or any other side constraints) as such. Their solution could be to pay the shopkeepers to change the choice architecture. If it could be proven that the benefits would outweigh the costs, there would be no need for further justifications. The question mark here is that aggregative utilitarianism is not universally accepted and is unlikely to survive the test of reflective equilibrium. It does not hurt, however, to keep this option in mind. Possible public measures include information guidance, subsidies, and regulation. Nudging was supposed to tally with the first and raise no issues of public spending and restrictions. If nudging arrangements cannot be made effective without straying from this ideal to the direction of regulations, why not consider monetary incentives, as well?

The most likely answer to the question is fear of socialism or reluctance to accept a fully fledged welfare state. The point could be pressed further, and a comprehensive welfare state defended, but anticipating objections in the reflective equilibrium process, we have, in Fig. 2, “switched off” the economic extremes, libertarianism, and socialism. What is left is the middle ground populated by compromise views such as the justice as fairness approach championed by Rawls (1972 [1971]) and the luck egalitarian or left libertarian take of Ronald Dworkin (1981a; 1981b) and Gerald Cohen (1989). The latter was brought to the limelight by Elizabeth Anderson (1999), who probably misinterpreted it (Ahola-Launonen 2018), but these debates often have a life of their own.

Although some care ethicists could condone nudging arrangements, both coercive and noncoercive, others can reject them as too calculating and atomistic. If our moral and political worth is defined collectively by the intersection of our memberships in overlooked and ignored groups, we may think that the recognition others owe us is absolute and nonnegotiable. This is not an ideal position to start a reflective equilibrium exercise, which presumes at least some flexibility in the views assessed. We have accounted for this in Fig. 2 by excluding (the darkened horizontal strips) categorical recognition ethics as well as aggregative utilitarianism, conservative communitarianism, technology-and-business-first enthusiasm, and the economic opposites, libertarianism, and socialism. The public consensus that we are looking for cannot probably be found in extremes. The empirical evidence there is for the public acceptance of *noncoercive nudging practices* (Hagman et al. 2015; Petrescu et al. 2016; Reisch et al. 2017; Schmidt and Engelen 2020) already indicates that the toleration is conditional upon shared ends and recognized authorities (Häußermann 2019). It stands to reason that even more background consensus is needed for *coercive nudging arrangements*.

As for the basis of such a consensus, we already suggested in setting our tasks that the legitimation of coercion could rely on beneficence, non-maleficence, autonomy, good practices, the protection of private property, or the reduction of alienation. These have their foundation in the key political moralities identified in our map of justice (Figs. 1 and 2). We have now ruled out libertarian and socialist concerns for private property and alienation insofar as they are offered as comprehensive foundations for public policies. Autonomy in its moderate forms is still in the toolbox, and so are promoting well-being and refraining from causing harm. Good practices, as solid constituents of a good and fair social life, are still on the agenda, but their precise content is what we are trying to define. Tradition as such, be it conservative or reformative, cannot be the sole guide.

Beneficence and nonmaleficence are principles used in professional biomedical ethics (Beauchamp and Childress 2019 [1979]). They have their place in that context, but their application to the shopkeepers’ plight is not without its hitches. Merchants are not professionals in the sense that physicians and other healthcare personnel are, and it is problematic to assign them a duty to watch over the well-being of their clients similar to the obligations of doctors and nurses (cf., however, Bowie 2009). Their customers do not approach them in a time of dire need, vulnerable to their machinations. As for nonmaleficence, we are back to asking the crucial question. Even if customers who buy junk food, according to popular understanding of their own free will, would suffer ill health as a long-term consequence, would the harm be inflicted by shopkeepers? The chain of causation is complicated and the legal principle “volenti non fit injuria” (the voluntary assumption of risk exonerates other parties) seems to apply. Does it, though? Is the choice voluntary and autonomous? Or does this provide a way of justifying the coercion, after all?

Let us go back to basics. As Sunstein (2015, 420–422) has argued, the existence of a choice architecture is inevitable even before any interventions occur. Shopkeepers do not operate in a vacuum—they give visibility to certain products and not others guided by some influences that already dominate their environment and social landscape. If they display junk food, this may be because the store chain they are linked with expects them to do so, or because the culture they live in favors the prevailing product placement, or because they believe that they can optimize their returns by doing what has been done before. It is not as if the governmental authorities were the original manipulators here. Their work for promoting public health has been preceded by the work of food companies and the advertising industry to (indirectly) deteriorate public health (Nestle 2000; Schor and Ford 2007; Royne and Levy 2008; Kearns et al. 2015; Kucharczuk et al. 2022).

This does not make shopkeepers culpable in the sense that they should be punished for their past deeds. But if the legislator now knows that merchants could (be made to) contribute to public health by their product placement, why would it be wrong to remind them and prompt them by regulations and incentives? Fig. 2 (the undarkened middle strip) indicates an area of relative consensus between otherwise conflicting views on justice. The middle views do not have to agree with one another on the higher-level axioms they apply—communitarians do not have to believe in the objective good of the many, utilitarians do not have to believe in spontaneously formed traditions, care and recognition ethicists do not have to believe in the universality of values, and capability thinkers do not have to believe in the importance of intersectional identities. Moderate representatives of these views only need to settle on the one lower-level norm that pertains to this issue, namely, “It is all right to promote public health by product-placement regulations or subsidies, when this can be done with high probability and reasonable cost.”

If they can agree on this notably neutral and unassuming practical rule, however, the way is clear for justifying other health-related interventions, as well. These would not have to be limited to noncoercive ones (in any sense), as the reflective-equilibrium-type consensus would provide a sufficient justification whatever the other particulars of the case. Suggestions by public authorities would be subjected to weighing, and proposals that raise too much theoretical opposition would be dropped. The ones that survive would have support from the practical consensus of the moderately minded. Not-so-moderately-minded opponents of coercion and subsidies would not be a part of the equation.

## Goodbye Libertarian Nudge Ideology, Hello Moderately Reformative Political Moralities!

Our considerations lead to two conclusions, one conceptual and the other ideological. First, as we (and others before us) have argued, there is no such thing as “libertarian paternalism.” Even nudging as a public practice would have to be disallowed by strict libertarians because it requires tax-based funding. Coercive nudging arrangements are even more rigidly out of the question, as they would be a direct interference with the freedom of (in our case) shopkeepers, who would be denied the right to control their private property and private business free from state intervention. Secondly, if (or since) we think that nudging practices and arrangements merit a justification, that justification has to be found in more moderate political moralities. These political moralities will not necessarily shy away from the use of coercion or incentives, and this widens the scope of legitimate public health interventions.

And so they should, even according to the founders of the nudge ideology. George Loewenstein and Nick Chater (2017) recently raised a concern that nudges are in danger of becoming a substitute to other forms of steering and policymaking. State authorities may come to believe that gentle changes in the choice architecture are all that is needed in public health promotion, with suboptimal results. In a response to this concern, Thaler (2017) partly agreed but pointed out that nudges were never meant to replace other forms of control, only to complement them, as evidenced by the first formulations of the ideology (Camerer et al. 2003; Thaler and Sunstein 2003). Our analysis endorses this intent and points out how it can be defended without employing the misleading epithet “libertarian paternalism” held so dear by Thaler (2017).

To reiterate the main point, nudges do not provide public health authorities with a way of interfering in people’s lives without interfering in people’s lives. However we define freedom, autonomy, people’s best interest, coercion, and other related concepts, steering by nudges has its expenses, not unlike those of many other public health interventions. Insofar as the nudge ideology has blurred this understanding, it should be restored and other alternatives brought to the fore again.

To refer to our map of justice (Figs. 1 and 2), we have suggested that a consensus on nudging and other interventions can best be found by concentrating on the non-economic dimensions of political moralities. In his compromise view on redistribution, Rawls (1972 [1971]) tried to settle the “vertical” disagreement between libertarians and socialists by suggesting a caring meritocracy. He paid less attention to the “horizontal” dimension, where collectivism (on the left in our map) and individualism (on the right) clash. Thaler and Sunstein’s fellow creators of the nudge approach, Colin Camerer, Samuel Issacharoff, George Loewenstein, Ted O’Donoghue, and Matthew Rabinarchitec (2003), hinted at a similar solution in their original contribution, titled “Regulation for conservatives.” We have tried to demonstrate that a wider consensus might be reached by including moderately reformative political moralities in the endeavor. This could be a way to produce a widely acceptable basis for both nudging arrangements and other measures for promoting public health.

## Data Availability

All data used in this study is in the public domain.
